# Wide Genetic Diversity of *Blastocystis* in White-Tailed Deer (*Odocoileus virginianus*) from Maryland, USA

**DOI:** 10.3390/microorganisms9061343

**Published:** 2021-06-21

**Authors:** Jenny G. Maloney, Yunah Jang, Aleksey Molokin, Nadja S. George, Monica Santin

**Affiliations:** Environmental Microbial and Food Safety Laboratory, Agricultural Research Service, United States Department of Agriculture, Beltsville, MD 20705, USA; jenny.maloney@usda.gov (J.G.M.); yunahj73@gmail.com (Y.J.); aleksey.molokin@usda.gov (A.M.); nadja.george@usda.gov (N.S.G.)

**Keywords:** *Blastocystis*, MinION, NGS, ribosomal RNA, subtypes, white-tailed deer, USA

## Abstract

*Blastocystis* is a gastrointestinal protist frequently reported in humans and animals worldwide. Wildlife populations, including deer, may serve as reservoirs of parasitic diseases for both humans and domestic animals, either through direct contact or through contamination of food or water resources. However, no studies of the occurrence and subtype distribution of *Blastocystis* in wildlife populations have been conducted in the United States. PCR and next generation amplicon sequencing were used to determine the occurrence and subtypes of *Blastocystis* in white-tailed deer (*Odocoileus virginianus*). *Blastocystis* was common, with 88.8% (71/80) of samples found to be positive. Twelve subtypes were identified, ten previously reported (ST1, ST3, ST4, ST10, ST14, ST21, and ST23–ST26) and two novel subtypes (ST30 and ST31). To confirm the validity of ST30 and ST31, MinION sequencing was used to obtain full-length *SSU* rRNA gene sequences, and phylogenetic and pairwise distance analyses were performed. ST10, ST14, and ST24 were the most commonly observed subtypes. Potentially zoonotic subtypes ST1, ST3, or ST4 were present in 8.5% of *Blastocystis*-positives. Mixed subtype infections were common (90.1% of *Blastocystis*-positives). This study is the first to subtype *Blastocystis* in white-tailed deer. White-tailed deer were found to be commonly infected/colonized with a wide diversity of subtypes, including two novel subtypes, zoonotic subtypes, and subtypes frequently reported in domestic animals. More studies in wildlife are needed to better understand their role in the transmission of *Blastocystis*.

## 1. Introduction

*Blastocystis* sp. is one of the most common protists colonizing/infecting the gastrointestinal tract of humans and numerous animals and has a global distribution [1,2,3,4]. The pathogenicity of *Blastocystis* is controversial, as the presence of *Blastocystis* in humans has been associated with gastrointestinal symptoms and/or urticaria, but it is also commonly found in asymptomatic individuals [5,6,7]. It has also been suggested that *Blastocystis* colonization could be associated with a healthy gut microbiome [4]. *Blastocystis* transmission is via the fecal-oral route, with direct transmission through contact with infected humans/animals or indirect transmission through ingestion of contaminated food and water [8,9,10,11]. The identification of *Blastocystis* in a broad range of animals, including pets, livestock, and wildlife, indicates that animals could be a potential source of infection for humans [3].

There is considerable genetic diversity among *Blastocystis* specimens isolated from mammals and birds that has been described based on polymorphisms in the small subunit ribosomal RNA (*SSU* rRNA) gene [12]. So far, 29 genetic variants, called subtypes (ST), have been proposed, and of those only 25 subtypes meet the current recommended criteria for unique subtype designations (ST1–ST17, ST21, ST23–ST29) [13,14,15,16]. Ten subtypes have been reported in humans, ST1–ST9 and ST12, with most studies reporting primarily ST1–ST4 [17]. The subtypes reported in humans have also been observed in animals indicating that these subtypes may have zoonotic potential [18,19,20,21,22]. For example, the identification of ST6, a subtype mostly identified in avian species, in slaughterhouse staff members provided evidence of the potential zoonotic transmission of this subtype through direct contact between chickens and their handlers [21]. Similarly, ST5, the most common subtype reported in pigs, is only sporadically reported in humans but was present in piggery staff, suggesting that close contact with pigs was associated with zoonotic transmission of ST5 in piggery workers [20].

The multiple transmission routes and the capacity of *Blastocystis* to infect many avian and mammalian hosts, including wild species, provide the appropriate conditions for transmission between humans and wild and domestic animals [3]. However, few studies on the occurrence and subtypes of *Blastocystis* in wildlife have been conducted, and there are no studies from the United States [3]. Better knowledge of subtypes of *Blastocystis* in wildlife is necessary to understand the potential role of wildlife in the transmission dynamics of this organism and the potential role of wildlife as reservoirs for human infection. The wild ungulate communities, including deer populations, frequently occur sympatrically with free-ranging domestic animals and are observed in close proximity to human populations. *Blastocystis* has been identified from a wide range of wild ungulates [3], but information on the presence and subtype distribution of *Blastocystis* in deer are limited (Table 1). Previous studies have reported the presence of *Blastocystis* in wild deer and in deer living in captivity and on farms with significant differences in occurrence between studies. To date, *Blastocystis* has been documented in 12 deer species, but animal numbers in these surveys tend to be small (Table 1). Furthermore, reported prevalence ranges widely from 0.8% in farmed sika deer in China [23] up to 100% in multiple populations including captive white-lipped deer (*Cervus albirostris*) in China [24], fallow deer from a zoo in Mauritius [25], captive marsh deer (*Blastocerus dichotomus*) and gray brocket (*Mazama gouazoubira*) in Brazil [26], and muntjac deer, rein deer, and red deer from a zoo in the United Kingdom [27,28]. There are no published reports of testing for *Blastocystis* in any deer species in the United States and no studies in white-tailed deer (*Odocoileus virginianus*) (WTD) worldwide. In the state of Maryland, the white-tailed deer population is estimated at 240,000 deer, with the population increasing on an average of 12% every 5 years, mostly due to the conversion of forested and agricultural lands into suburban areas that improve habitat conditions for deer (MD-Annual-Deer-Report-2019-2020.pdf (maryland.gov); accessed on 4 May 2021). WTD populations in Maryland live near or in suburban areas where they frequently encounter humans, companion animals, and livestock. The present study was conducted to provide the first examination of the presence of *Blastocystis* in WTD from Maryland (USA) and to characterize subtypes present in samples using next generation amplicon sequencing (NGS). Additionally, Oxford Nanopore MinION long-read sequencing was used to generate the full-length *SSU* rRNA gene of two novel *Blastocystis* sequences identified in WTD to confirm the validity of these sequences as novel subtypes.

## 2. Materials and Methods

### 2.1. Source and Collection of Specimens

Feces were collected from 80 hunter-killed WTD during a managed hunt in Howard County, Maryland. The hunt was conducted under the supervision of county officials as part of a wildlife management program. The goals of the management program are to ensure the present and future well-being of deer and their habitat and to maintain the deer population at a level that safeguards compatibility with human land uses and natural communities. Fecal specimens were collected over two hunting seasons: 2010–2011 (*n* = 52) and 2011–2012 (*n* = 28). Age and gender data were recorded for each WTD sampled (Table 2). A fecal specimen was collected from each animal directly from the rectum into a plastic cup. Cups were capped, labeled, and immediately placed in an insulated container packed with ice or cold packs. Specimens were transported to the USDA laboratory in Beltsville, Maryland and processed within 1–3 days of collection.

### 2.2. Parasite Concentration from Feces and DNA Extraction

To concentrate parasites, fecal specimens were sieved and subjected to CsCl density centrifugation, as previously described [35]. DNA was extracted from each CsCl-concentrated fecal sample using the DNeasy Tissue Kit (Qiagen, Valencia, CA, USA) as directed by the manufacturer with minor modifications. In brief, a 50 μL suspension of each CsCl-concentrated fecal sample was suspended in 180 μL of ATL buffer and thoroughly mixed. Twenty μL of proteinase K (20 mg/mL) was added to this suspension, and the mixture was incubated at 55 °C overnight. Then 200 μL of AL buffer was added, and DNA was purified per manufacturer’s instructions and eluted in 100 μL of AE buffer.

### 2.3. Molecular Detection and Subtype Identification Using Next Generation Amplicon Sequencing

A next generation amplicon sequencing strategy was used to detect *Blastocystis* as previously described [36]. Briefly, a PCR using primers ILMN_Blast505_532F and ILMN_Blast998_1017R was used to screen all 80 WTD samples. These primers amplify a fragment of the *SSU* rRNA gene (ca. 500 bp) and are identical to Blast505_532F/Blast998_1017R [37], with the exception of containing the Illumina overhang adapter sequences on the 5′ end. Final libraries were quantified by Qubit fluorometric quantitation (Invitrogen, Carlsbad, CA, USA) prior to normalization. A final pooled library concentration of 8 pM with 20% PhiX control was sequenced using Illumina MiSeq 600 cycle v3 chemistry (Illumina, San Diego, CA, USA). Paired end reads were processed and analyzed with an in-house pipeline that uses the BBTools package v38.82 [38], VSEARCH v2.15.1 [39], and BLAST+ 2.11.0 [40]. Briefly, read pairs were merged, filtered for quality and length, denoised, and checked for chimeric sequences. Clustering and the assignment of centroid sequences to operational taxonomic units (OTU) was performed within each sample at a 98% identity threshold. Only those OTUs with a minimum of 100 sequences were retained and then checked for chimeras once more. OTUs were then blasted against *Blastocystis* references from NCBI. All hits below an alignment length of 400 bp were removed. All raw fastq files were deposited to the NCBI sequence read archive under accession numbers SRR14607063–SRR14607133. The nucleotide sequences generated using NGS in this study were deposited in GenBank under the accession numbers MZ267636–MZ267676.

### 2.4. PCR Amplification and Sequencing of the Full-Length SSU rRNA Gene

For four WTD samples (#22, #27, #73, #79) containing novel subtypes (ST30 and ST31), we used a previously described Nanopore sequencing strategy to generate the approximately 1800 base pair *SSU* rRNA gene [41]. Briefly, a PCR using SSU-F1 (5′-AAC CTG GTT GAT CCT GCC AGT AGT C-3′) and SSU-R1 (5′-TGA TCC TTC TGC AGG TTC ACC TAC G-3′), which amplify the *SSU* rRNA gene of most eukaryotic organisms, was performed [42]. Each reaction used 1 µM forward and reverse primers and 12.5 µL of KAPA HiFi HotStart ReadyMix (KAPABioSystems, Cape Town, South Africa) in a 25 µL reaction volume. Initial denaturation was performed at 98 °C for 5 min followed by 35 cycles of amplification (20 s at 98 °C, 45 s at 60 °C, and 90 s at 72 °C) and final extension for 5 min at 72 °C. Following amplification, amplicons were visualized using a QIAxcel (Qiagen, Valencia, CA, USA) and quantified using a Qubit fluorometer (ThermoFisher Scientific, Waltham, MA, USA).

The Nanopore sequencing library was prepared using the Oxford Nanopore Technologies (ONT) SQK-LSK109 Ligation Sequencing Kit (ONT, Oxford, UK) following the manufacturer’s protocol for Amplicons by Ligation (ACDE_9064_v109_revQ_14Aug2019). Amplicons were quantified and diluted to ensure 150 fmol of DNA was used as input into library prep as recommended by the protocol. The nanopore library was run on an R9.4 flow cell (FLO-MIN106) using an ONT MinION Mk1B and MinKNOW v20.06.15 software (ONT, Oxford, UK). Basecalling was performed using Guppy v4.0.11 (ONT, Oxford, UK) using a minimum quality score cut off of 7 for filtering low quality reads. FASTQ reads were also length filtered to only include reads between 1700 and 2000 nucleotides. Reads were then corrected using Canu v2.1 [43] and consensus sequences were generated by clustering reads using the vsearch–cluster_fast command (vsearch v2.14.1) with a 98% identity threshold, checked for chimeras, and polished as previously described [40].

For comparison purposes, full-length sequences and partial sequences obtained with MinION and MiSeq, respectively, were aligned using ClustalW in MegAlign 15 (DNASTAR Lasergene 15, Madison, WI, USA), and pairwise distances between consensus sequences were calculated. The full-length nucleotide sequences generated in this study were deposited in GenBank under the accession numbers MZ267674–MZ267679.

### 2.5. Phylogenetic and Pairwise Distance Analysis

The full-length *SSU* rRNA gene nucleotide sequences obtained in this study, appropriate full-length *Blastocystis* reference nucleotide sequences obtained from the reference database found at http://entamoeba.lshtm.ac.uk/blastorefseqs.htm (accessed on 4 May 2021), as well as other full-length sequences available in GenBank to include all currently accepted subtypes were aligned to generate a phylogenetic tree which was rooted using *Proteromonas lacertae*, a Stramenopile which is closely related to *Blastocystis*, as an outgroup. Nucleotide sequences were aligned with the Clustal W algorithm and the phylogenetic analysis was performed using the Neighbor-Joining (NJ) method, and genetic distances calculated with the Kimura 2-parameter model using MEGA X [44,45]. A total of 1950 positions were included in the final dataset, which included 70 nucleotide sequences. Bootstrapping with 1000 replicates was used to determine support for the clades generated. Additionally, evolutionary analysis was conducted to establish divergence between nucleotide sequences (pairwise distance) using the Kimura 2-parameter model in MEGA X.

Furthermore, for comparative purposes, identical phylogenetic and pairwise distance analyses using the same 70 nucleotide sequences utilized for full-length analyses were conducted for the regions of the *SSU* rRNA gene amplified by the two most common standard primers sets used for *Blastocystis* in survey studies to amplify and sequence the regions known as barcoding and Santin [37,46]. There was a total of 590 and 570 positions in the final datasets for barcoding and Santin regions, respectively.

## 3. Results

### 3.1. Prevalence of Blastocystis in White-Tailed Deer

Of the 80 WTD fecal samples tested in this study, 71 (88.8%) were determined to be positive for *Blastocystis* by PCR. Positive-*Blastocystis* samples were observed in all age groups (Table 2). The highest prevalence was found in fawns (100%; 3/3) follow by adults (88.9%; 48/54) and yearlings (86.4%; 19/22). *Blastocystis* was detected at similar levels in males (86.1%; 31/36) and females (90.7%; 39/43).

### 3.2. Subtypes of Blastocystis in White-Tailed Deer

The 71 *Blastocystis*-positive samples generated a total of 13,785,248 paired end reads. Following end trimming, quality filtering, and pair merging, 5,006,876 reads remained. The removal of chimeric sequences left 3,955,512 merged reads, which were used for OTU generation. Clustering generated 230 OTUs that aligned to *Blastocystis* among the 71 samples, and of those 38 were unique *Blastocystis* sequences (Table 3).

Twelve subtypes were identified in *Blastocystis*-positive WTD, ten previously reported subtypes ST1, ST3, ST4, ST10, ST14, ST21, ST23, ST24, ST25, ST26, and two novel subtypes named ST30 and ST31 (Table 2 and Table 3). The most frequently observed subtypes in this study were ST10 and ST24 found in 71.8% (51/71) and 77.5% (55/71) of *Blastocystis*-positive WTD, respectively (Table 3). ST14 was the third most abundant subtype and was detected in 42.3% (30/71) of the *Blastocystis*-positive WTD. Novel subtypes ST30 and ST31 were frequently observed and were detected in 15.5% (11/71) and 26.8% (19/71) of the *Blastocystis*-positive WTD, respectively (Table 3). Potentially zoonotic subtypes ST1, ST3, and ST4 were observed in one, two, and three samples, respectively (Table 3). An increase in the number of subtypes associated with age was observed, with six subtypes in fawns, eight in yearlings, and 11 in adults (Table 2). Eleven and eight subtypes were identified in males and females, respectively (Table 2).

**Figure 1:**
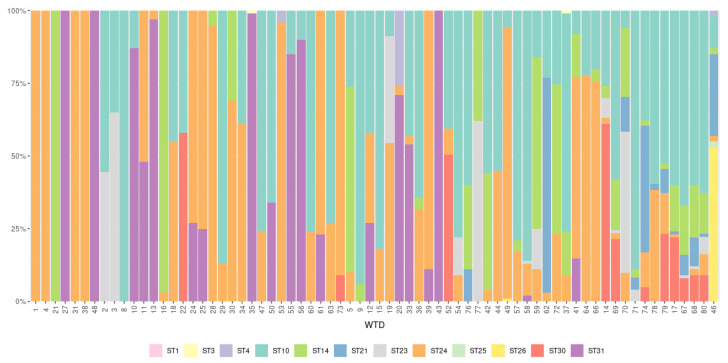
Relative abundance of reads (%) of each subtype present in the 71 *Blastocystis*-positive white-tailed deer (WTD) samples arranged by number of subtypes present from lowest to highest.

Mixed infections with two or more *Blastocystis* subtypes were frequently observed. Infections with more than one subtype were identified in 90.1% (64/71) of *Blastocystis*-positive WTD, while single infections were only detected in 9.9% (7/71) of *Blastocystis*-positive WTD. Only three subtypes were present as single infections, ST14, ST24, and ST31, which were found in 1.4% (1/71), 5.6% (4/71), and 2.8% (2/71) of the *Blastocystis*-positive WTD, respectively (Table 2; Figure 1). Thirty combinations of subtypes were observed in *Blastocystis*-positive samples including co-infection with two, three, four, five, six, and seven subtypes found in ten, ten, five, three, one, and one samples respectively (Table 2). The relative abundance of subtypes present in each of the 71 *Blastocystis*-positive samples varied widely (Figure 1). Co-infection with ST10/ST24 and ST10/ST14/ST24 were the most common mixed subtype combinations observed, and they represented 12.5% (8/64) and 10.9% (7/64) of mixed subtype infections, respectively.

### 3.3. Blastocystis Intra-Subtype Variation

Thirty-eight unique *Blastocystis* sequences were identified in the 71 *Blastocystis*-positive WTD (Table 3). High intra-subtype variation was observed for ST10 with 14 unique sequences among the 51 ST10-positive WTD. Intra-subtype variation was lower for the rest of the subtypes identified in WTD, with three unique sequences for ST14, ST21, ST24, ST26, and ST30, two unique sequences for ST1, ST3, and ST4, and a single unique sequence for ST23, ST25, and ST31 (Table 3).

### 3.4. Validation of Novel Subtypes ST30 and ST31

Nucleotide sequences for the novel subtypes generated by Illumina were compared to nucleotide sequences available in the GenBank database. The closest match to *Blastocystis* sequences available in GenBank for ST30 was 99.8%–100% to unpublished *Blastocystis* nucleotide sequences with no subtype information that were obtained from sheep fecal samples from Belgium (HF569206). For ST31, the closest nucleotide sequences available in GenBank were three unpublished *Blastocystis* nucleotide sequences also without subtype information and that were obtained from fecal samples of Korean water deer from South Korea (MT114839, MT114842, MT114845) with 97.7% similarity. To confirm the validity of the novel subtypes according to recently proposed guidelines, we used a Nanopore sequencing strategy to obtain the near full-length nucleotide sequence of the *SSU* rRNA gene using DNA from four WTD (#22, #27, #73, #79) positive for novel subtype sequence variants. Full-length sequences were successfully obtained for both novel subtypes and for all three variants of ST30. Full-length nucleotide sequences of the *SSU* rRNA gene for the three sequence variants of ST30 were obtained from WTD#22, WTD#73, and WTD#79, while ST31 was obtained from WTD#27. Additionally, full-length sequences were generated for other subtypes present in WTD#22 and WTD#79. A full-length ST10 sequence was obtained from WTD#22, and from WTD#79, individual sequences for ST10, ST21, and ST24 were obtained. ST21 and ST24 full-length sequences from WTD#79 have been previously published (MW887929 and MW887930) [16]. There was 100% agreement between the Illumina sequence and the same region within the MinION sequence for all sequences of ST30 and ST31.

**Figure 2:**
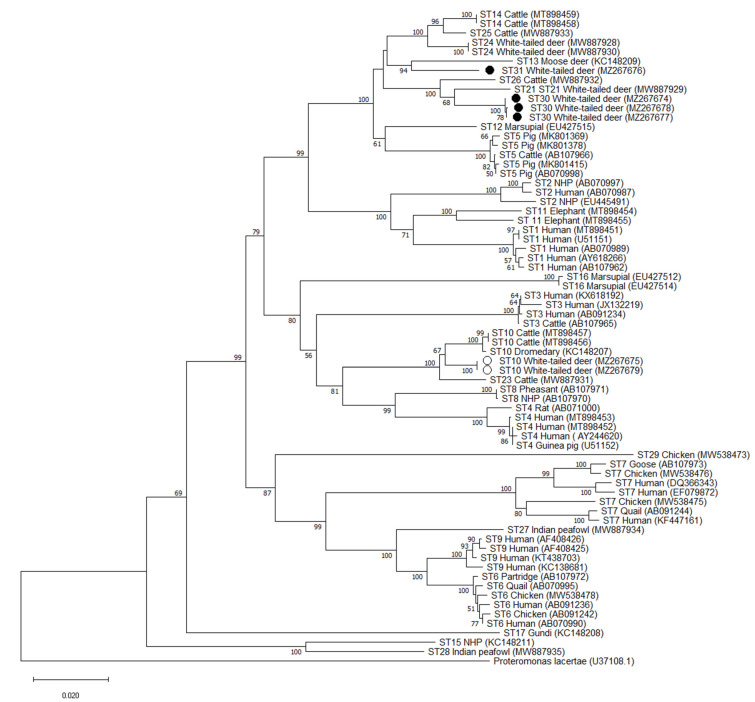
Phylogenetic relationships among *Blastocystis* full-length sequences generated in the present study (novel subtypes are represented with a black filled circle and other subtypes with an unfilled circle) and representative reference sequences of all accepted subtypes. *Proteromonas lacertae* was used as outgroup taxon to root the tree. Analysis was conducted by a neighbor-joining method. Genetic distances were calculated using the Kimura two-parameter model. This analysis involved 70 nucleotide sequences, and there were a total of 1950 positions in the final dataset. Bootstrap values lower than 50% are not displayed.

**Figure 3:**
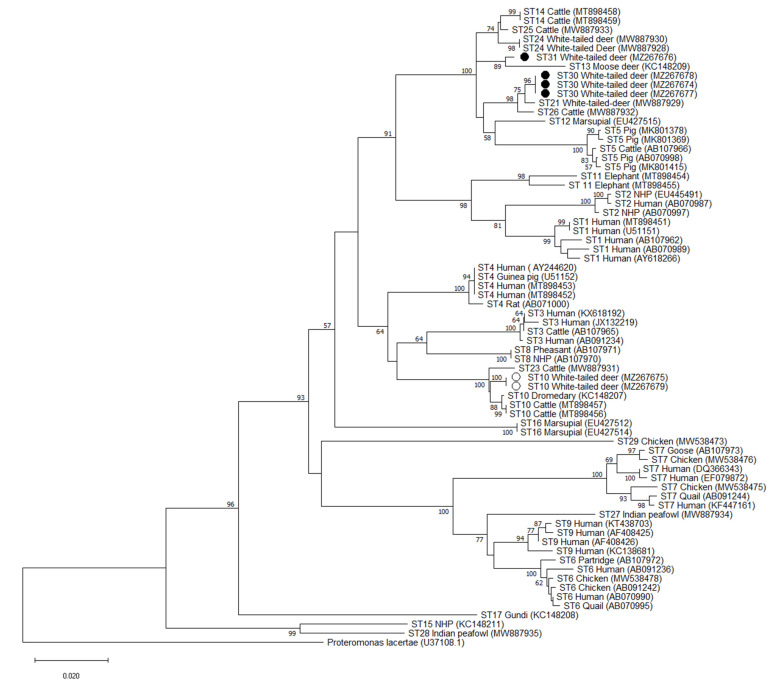
Phylogenetic relationships among *Blastocystis* barcoding region sequences generated in the present study (novel subtypes are represented with a black filled circle and other subtypes with an unfilled circle) and representative reference sequences of all accepted subtypes. *Proteromonas lacertae* was used as outgroup taxon to root the tree. Analysis was conducted by a neighbor-joining method. Genetic distances were calculated using the Kimura two-parameter model. This analysis involved 70 nucleotide sequences, and there were a total of 590 positions in the final dataset. Bootstrap values lower than 50% are not displayed.

**Figure 4:**
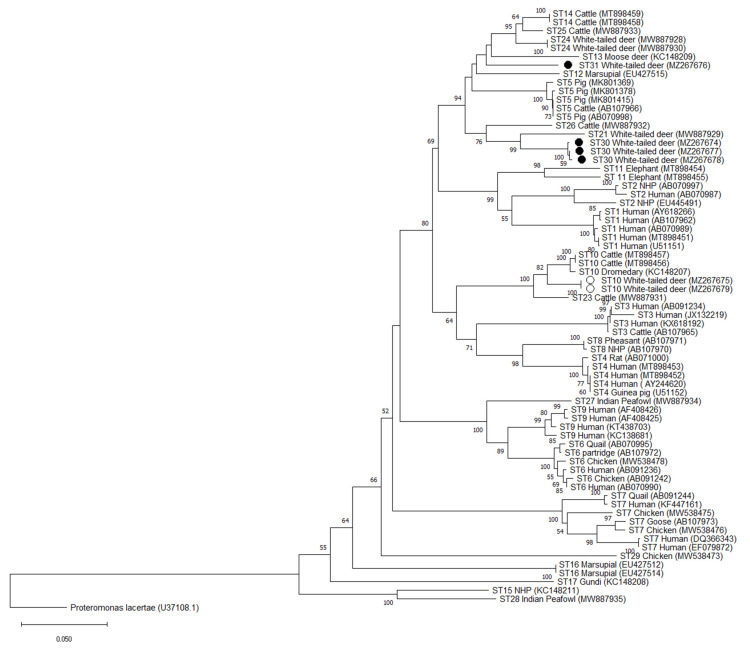
Phylogenetic relationships among *Blastocystis* Santin region sequences generated in the present study (novel subtypes represented are with a black filled circle and other subtypes with an unfilled circle) and representative reference sequences of all accepted subtypes. *Proteromonas lacertae* was used as outgroup taxon to root the tree. Analysis was conducted using a neighbor-joining method. Genetic distances were calculated using the Kimura two-parameter model. This analysis involved 70 nucleotide sequences, and there were a total of 571 positions in the final dataset. Bootstrap values lower than 50% are not displayed.

Phylogenetic analysis of full-length sequences using the NJ method demonstrated that all 3 variants of the ST30 cluster with ST21 and ST26 with bootstrap support of 68 for the cluster formed by ST30 and ST21, and bootstrap support of 100 for the cluster formed by ST30/ST21 and ST26 (Figure 2). Similar clustering is observed at the barcoding and Santin regions, with ST30 clustering with ST21 and ST26. Bootstrap values of 75 and 99 were observed for the cluster formed by ST30 and ST21 and 98 and 76 for ST30/ST21 and ST26 for the Barcoding and Santin regions, respectively (Figure 3 and Figure 4). For ST31, phylogenetic analysis using full-length sequences showed ST31 clustering with ST13 with bootstrap support of 94 (Figure 2). Similar clustering is observed using the barcoding region with bootstrap support of 89 (Figure 3). For the Santin region, ST31 no longer forms a clade with ST13, but does cluster within a clade formed by ST12, ST13, ST14, ST24, and ST25 (Figure 4).

Pairwise distance comparisons were used to evaluate the percentage of shared sequence identity of ST30 and ST31 with known subtypes using full-length sequences, the barcoding region, and the Santin region (Supplementary Tables S1–S3). Using full-length sequences, the highest percentage of sequence similarity for ST30 was 97% with ST21 and ST26, while ST31 shared 95% sequence similarity with ST12, ST13, ST14, ST24, and ST25 (Supplementary Table S1). Sequence similarity was higher in the barcoding region, with 99% sequence similarity between ST30 and ST21 and ST26 and 98% similarity between ST31 and ST13, ST14, ST24, ST25, ST21, and ST30 (Supplementary Table S2). On the other hand, pairwise distance comparisons for novel subtypes in the Santin region exhibited greater degrees of divergence than those obtained by analysis of full-length sequences. In the Santin region the highest sequence similarity for ST30 was 93% with ST21, while ST31 exhibited a 93% similarity with ST24 and ST25 (Supplementary Table S3).

## 4. Discussion

*Blastocystis* is a common parasite of humans, which is also frequently observed in wild and domestic animals [3]. However, the role of wildlife in *Blastocystis* transmission is not well explored, especially in the United States. Deer are a common wildlife species with habitats that overlap with humans and other domestic and wild animals, which creates the potential for deer to act as reservoirs for pathogens among these populations. Yet, no studies of *Blastocystis* prevalence or subtype distribution have been conducted in WTD. In the present study, WTD from Maryland, USA were tested for the presence of *Blastocystis* by PCR and next generation amplicon sequencing, and WTD were found to be commonly infected/colonized with multiple subtypes of this parasite.

*Blastocystis* was observed in 88.8% (71/80) of the WTD included in this study, indicating that *Blastocystis* is a common parasite of WTD. Furthermore, *Blastocystis* occurrence was high in all gender and age categories measured (Table 2). The number of large studies on *Blastocystis* occurrence in deer are limited, and only two studies have measured *Blastocystis* occurrence in wild deer populations (Table 1). Of the two previous studies which surveyed *Blastocystis* in wild deer populations, occurrences of 2% and 41% were reported [29,30]. These studies were conducted in Australia in red deer [30] and in South Korea in Korean water deer [29], which may contribute to the large difference in occurrence between these studies and the higher occurrence of *Blastocytsis* in WTD observed in this study. All three studies have targeted different deer species in different geographic locations and used different detection methods to test for the presence of *Blastocystis*. The high occurrence of *Blastocystis* in WTD from Maryland, USA and in deer in general indicates that more studies in deer from other regions of the USA and worldwide are needed to better characterize *Blastocystis* occurrence in deer.

A wide diversity of subtypes was observed in WTD in this study. There were 12 subtypes and 38 unique *Blastocystis* nucleotide sequences among the 71 *Blastocystis*-positive WTD (Table 2 and Table 3). Ten of the subtypes observed in WTD in this study (ST1, ST3, ST4, ST10, ST14, ST21, ST23, ST24, ST25, and ST26) are previously established subtypes [13,16]. Of these, only four subtypes (ST1, ST4, ST10, and ST14) have been previously reported in other studies of deer from around the world (Table 1). This is the first study to report ST3 in deer expanding the host range of this subtype. Although ST3 was only observed in two samples, representing 2.8% of *Blastocystis*-positive deer, its presence in deer is particularly notable as ST3 is the most common subtype reported in humans [47]. In fact, potentially zoonotic subtypes were present in 8.5% (6/71) of *Blastocystis*-positive WTD. The presence of ST3 and two other potentially zoonotic subtypes, ST1 and ST4, in WTD could indicate the potential for zoonosis as well as the possibility of reverse zoonosis between WTD and humans.

Subtypes ST10 and ST14 are two of the most commonly reported subtypes in ruminants (Table 1) and are commonly reported in other studies of deer [3]. ST10 and ST14 were the second and third most common subtypes observed in WTD in 71.8% and 42.3% of *Blastocystis*-positive WTD, respectively. The presence of these two subtypes in WTD further confirms the suitability of ruminants as hosts of ST10 and ST14. To our knowledge, this is the first study to report ST21, ST23, ST24, ST25, and ST26 in deer expanding their host range. These subtypes have been reported in studies of other wild and domestic animals and appear to be prevalent in ruminants [14,15,24,25,32,48,49,50]. The frequent occurrence of these subtypes in WTD in this study further supports the contention that ruminants may be common hosts of these subtypes. Interestingly, ST24 was observed in 77.5% of *Blastocystis*-positive WTD making in the most commonly observed subtype in this study. The high occurrence of ST24 in WTD in this study could indicate deer as a potential source of infection for domestic ruminants such as cattle which have lower reported occurrence of ST24 [36,48].

The presence of multiple subtypes of *Blastocystis* in individual WTD was very common in the study population. Mixed subtype infections were observed in 90.1% (64/71) of *Blastocystis*-positive WTD in 30 different combinations (Figure 1, Table 2). In the only other study to apply NGS to explore *Blastocystis* subtype diversity in ruminants, mixed infections were also abundant and observed in 65.3% (49/75) of *Blastocystis*-positive cattle [48]. NGS has also been used to explore *Blastocystis* subtype diversity in wild carnivores, humans, captive wild and domestic birds, chickens, wild boars, and pigs with mixed infections reported in 50%, 13.7%, 62.5%, 63.6%, 23.1%, and 15.4% of *Blastocystis*-positive hosts, respectively [7,14,15,51,52]. Thus, the occurrence of mixed subtype infection in WTD reported here is the highest in any *Blastocystis* host to date. Whether this finding is attributable to WTD physiology or ecology remains to be defined. However, the high occurrence of mixed infections, the high occurrence of *Blastocystis* overall, and the large number of subtypes present in this wild WTD population could indicate that exposure to multiple sources of infection contribute to infection risk and intra-subtype variability within individual hosts. While lower rates of mixed subtype infections and less subtype diversity in domestic and captive wildlife could indicate shared sources of infection within those populations [7,14,15,51,52].

Multiple sequence variants were observed for nine of the 12 subtypes reported in WTD (Table 3). However, ST10 presented with a markedly large degree of intra-subtype variability compared with other subtypes observed in WTD in this study (Table 3). There were 14 unique sequence variants of ST10, while all other subtypes had between one and three sequence variants. The intra-subtype variability observed in ST10 in WTD is similar to that reported using NGS in cattle where 11 unique ST10 sequence variants were observed among 15 *Blastocystis*-positive cattle [36]. Unlike cattle, WTD had far less intra-subtype diversity in ST14 and ST24 than might be expected given the common occurrence of these subtypes in WTD. In cattle, there were five unique sequence variants of ST14 reported among 16 positive samples and six unique sequence variants of ST24 among 14 positive samples [36]. While *Blastocystis*-positive WTD had only three unique sequence variants of ST14 among 30 positive samples and three unique sequence variants of ST24 among 55 positive samples. It is intriguing to speculate that the lack of variability in these two common subtypes of WTD could indicate some degree of host specificity of these sequence variants. Indeed, the three unique sequence variants of ST14 observed in WTD in this study share 99–100% sequence identity with an ST14 sequence originally reported in a mouflon (Genbank accession# KC148206) [25]. However, such a conclusion could only be drawn after more extensive sampling and comparison between other domestic and wild ruminant hosts of these subtypes.

There were two novel subtypes observed in WTD in this study, which we propose naming ST30 and ST31. Both novel subtypes were frequently observed, with ST30 in 15.5% (11/71) of *Blastocystis*-positive WTD and ST31 in 26.8% (19/71) of *Blastocystis*-positive WTD by NGS (Table 3). The NGS protocol used in this study generates sequences of approximately 500 base pairs of a region of the *SSU* rRNA gene. Recently-proposed guidelines suggest new subtype designations be based on nearly full-length *SSU* rRNA gene sequences [13]. To achieve full-length sequences for the two novel subtypes observed in this study, we employed a MinION sequencing strategy which has been demonstrated to be suitable for obtaining high quality full-length *Blastocystis* reference sequences [15,16,41]. Full-length sequences were obtained for all three sequence variants of ST30 and the single variant of ST31 observed using NGS (Table 3). These sequences were compared to other full-length reference sequences from accepted subtypes of *Blastocystis* (ST1–ST17, ST21, ST23–ST29) to determine if phylogenetic analysis and pairwise sequence comparison support their designation as new subtypes. Phylogenetic analysis demonstrated strong support for the branching of ST31 with bootstrap support of 94 (Figure 2). Pairwise comparison of full-length sequences also demonstrated that ST31 shares ≤ 95% sequence similarity with any known subtype (Supplementary Table S1). As such, ST31 clearly meets all recommended criteria for a new subtype designation. ST30 formed a clade with ST21 and ST26, where ST26 branches basally to ST21 and ST30, however, branching within the clade formed by ST21 and ST30 has bootstrap support of 68. Furthermore, pairwise comparisons for ST30 indicate it shares 97% sequence similarity with ST21 and ST26 (Supplementary Table S1). As ST30 does not clearly fall into any existing subtype category, we suggest it be given a novel subtype designation. This conclusion is further supported by the high degree of sequence variance observed for this subtype in the Santin region, where it varies from any named subtype by ≤7% (Supplementary Table S3). Lastly, NGS sequences of ST30 are a near 100% match to unpublished sequences with no subtype information from sheep from Belgium (Genbank accession #s HF569206, HF569208, HF569214, and HF569226) indicating that this subtype is found in multiple hosts and geographic regions. There is a clear advantage to providing novel subtypes with a designation that will allow researchers to easily and accurately subtype isolates to understand host specificity and epidemiology.

## 5. Conclusions

This study is the first to use NGS to characterize *Blastocystis* subtype diversity and occurrence in WTD and the first to study *Blastocystis* in WTD overall. The common occurrence of *Blastocystis* in WTD, coupled with the observation of a remarkable number of subtypes, mixed subtype infections, novel subtypes, and zoonotic subtypes in the study population, indicates that WTD and deer in general are an understudied population with potentially important roles in *Blastocystis* transmission to humans and domestic animals. More studies in deer and other wildlife populations from other regions of the United States and world are needed to understand the role of wildlife in *Blastocystis* transmission and epidemiology.

## Figures and Tables

**Table 1:** Summary of studies reporting prevalence and subtypes of *Blastocystis* identified in deer. Potentially zoonotic subtypes are in bold.

Host (Scientific Name)	Country	Source of Deer	No. of Samples Examined/ No. of Positives (%)	Subtype(s)	References
Eurasia elk (*Alces alces*)	United Kingdom	Zoo	2/1 (50%) ^a^	**ST4**(1), ST10(1), ST14(1), ND(1)	[27]
	United Kingdom	Zoo	3/1 (33.3%) ^a^	**ST4(1)**, ST10(1), ST14(4)	[28]
Fallow deer (*Dama dama*)	China	Captive	2/1 (50%)	ST10(1)	[24]
	Mauritius	Zoo	2/2 (100%)	ST10(2)	[25]
Gray brocket (*Mazama gouazoubira*)	Brazil	Captive	1/1 (100%)	**ST5**(1)	[26]
Korean water deer (*Hydropotes inermis argyropus*)	South Korea	Wild	125/51 (40.8%)	**ST4(1)**, ST14(25) ^b^	[29]
Marsh deer (*Blastocerus dichotomus*)	Brazil	Captive	1/1 (100%)	ND(1) ^b^	[26]
Muntjac deer (*Muntiacus reevesi*)	United Kingdom	Zoo	1/1(100%)	ST14(1)	[27]
	United Kingdom	Zoo	1/1(100%)	ST13(1)	[28]
Red deer (*Cervus elaphus*)	Australia	Wild	50/1 (2%)	**ST4**(1)	[30]
	China	Captive	3/1 (33.3%)	ST10(1)	[24]
	China	Zoo	5/2 (40%)	ST10(2)	[31]
	United Kingdom	Zoo	1/1(100%) ^a^	**ST4**(2), ST10(6)	[27]
	United Kingdom	Zoo	3/1(33.3%) ^a^	**ST4(3)**, ST10(5)	[28]
Reindeer (*Rangifer tarandus*)	China	Farm	104/7 (6.7%)	ST10(3), ST13(4)	[23]
	United Kingdom	Zoo	1/1(100%)	ST10(1)	[28]
Roe deer (*Capreolus capreolus*)	Denmark	Zoo	1 ^c^	ST10(1)	[32]
	United Kingdom	Zoo	2/1 (50%)	**ST5**(1)	[25]
Sika deer (*Cervus nippon*)	China	Farm	6/760 (0.8%)	ST10(5), ST14(1)	[33]
	China	Captive	8/3 (37.5%)	ST10(3)	[24]
	China	Farm	82/12 (14.6%)	ST10(10), ST14(2)	[23]
	China	Zoo	11/1 (9.1%)	**ST1(1)**	[4]
Spotted deer (*Axis axis*)	Bangladesh	Zoo	30/1 (3.3%)	ST14(1)	[34]
White-lipped deer (*Cervus albirostris*)	China	Captive	1/1 (100%)	ST10(1)	[24]
White tailed-deer (*Odocoileus virginianus*)	United States	Wild	80/71 (88.8)	**ST1(1)**, **ST3(2)**, **ST4(3)**, ST10(51), ST14(30), ST21(14), ST23(14), ST24(55), ST25(1), ST26(2), ST30(11), ST31(19) ^d^	This study

ND: Not determined. ^a^ The number of STs identified is based on the number of sequence positive clones obtained and does not correspond with the number of positive samples identified. ^b^ Not all PCR positive samples were successfully sequenced. ^c^ This study was not a survey. ^d^ The numbers of STs exceed the number of samples because multiple subtypes were observed in individual samples. For details of ST combinations, see Table 2.

**Table 2:** *Blastocystis* prevalence and subtypes observed in white-tailed deer (WTD) in Maryland, USA by age and gender. Potentially zoonotic subtypes are in bold.

		No. of WTD	No. *Blastocystis* Positives (%)	Subtypes Identified	Subtypes Combinations Observed in Individual Samples
Age group ^a^	Fawn	3	3 (100)	ST10, ST14, ST21, ST23, ST24, ST30	ST10/ST14/ST21(1); ST14/ST21/ST23(1); ST10/ST14/ST21/ST23/ST24/ST30(1)
	Yearling	22	19 (86.4)	**ST3**, ST10, ST14, ST23, ST24, ST26, ST30, ST31	**ST3**/ST31(1); **ST3**/ST10/ST14/ST24(1); ST10/ST14/ST24(2); ST10/ST24(4); ST10/ST24/ST26(1); ST10/ST23/ST24(1); ST10/ST30(1); ST10/ST31(2); ST10/ST24/ST31(2); ST14/ST24(2); ST24(1); ST24/ST31(1)
	Adult	54	48 (88.9)	**ST1**, **ST4**, ST10, ST14, ST21, ST23, ST24, ST25, ST26, ST30, ST31	**ST1**/ST10/ST21/ST24(1); **ST4**/ST24(1); **ST4**/ST24/ST31(1); **ST4**/ST10/ST14/ST21/ST24/ST25/ST26(1); ST10/ST14(2); ST10/ST14/ST23/ST24(1); ST10/ST14/ST24(5); ST10/ST14/ST31 (1); ST10/ST14/ST21/ST24/ST30(3); ST10/ST14/ST21/ST23/ST24(2); ST10/ST14/ST21/ST23/ST24/ST30(2); ST10/ST14/ST23/ST24/ST30(2); ST10/ST14/ST24/ST31(1); ST10/ST31(2); ST10/ST21/ST24(2); ST10/ST23(2); ST10/ST23/ST24(1); ST10/ST24(4); ST10/ST24/ST30(1); ST10/ST24/ST31(1); ST14(1); ST14/ST24(1); ST24(3); ST24/ST30(1); ST24/ST31(4); ST31(2)
Gender ^a^	Males	36	31 (86.1)	**ST3**, **ST4**, ST10, ST14, ST21, ST23, ST24, ST25, ST26, ST30, ST31	**ST3**/ST31(1); **ST3**/ST10/ST14/ST24(1); **ST4**/ST24(1); **ST4**/ST24/ST31(1); **ST4**/ST10/ST14/ST21/ST24/ST25/ST26(1); ST10/ST14/ST23/ST24(2); ST10/ST14/ST24(4); ST10/ST14/ST21/ST24/ST30(1); ST10/ST14/ST21/ST23/ST24(1); ST10/ST14/ST23/ST24/ST30(1); ST10/ST14/ST24/ST31(1); ST14/ST21/ST23(1); ST10/ST30(1); ST10/ST31(1); ST10/ST23(1); ST10/ST24(3); ST10/ST24/ST26(1); ST10/ST24/ST31(2); ST14/ST24(2); ST24(2); ST24/ST30(1); ST24/ST31(2)
	Females	43	39 (90.7)	**ST1**, ST10, ST14, ST21, ST23, ST24, ST30, ST31	**ST1**/ST10/ST21/ST24(1); ST10/ST14(2); ST10/ST14/ST21(1); ST10/ST14/ST24(3); ST10/ST14/ST31 (1); ST10/ST14/ST21/ST24/ST30(2); ST10/ST14/ST21/ST23/ST24(1); ST10/ST14/ST21/ST23/ST24/ST30(3); ST10/ST14/ST23/ST24/ST30(1); ST10/ST31(3); ST10/ST21/ST24(2); ST10/ST23(1); ST10/ST23/ST24(2); ST10/ST24(5); ST10/ST24/ST30(1); ST10/ST24/ST31(1); ST14(1); ST14/ST24(1); ST24(2); ST24/ST31(3); ST31(2)
Total		80	71 (88.8)	ST1, ST3, ST4, ST10, ST14, ST21, ST23, ST24, ST25, ST26, ST30, and ST31	**ST1**/ST10/ST21/ST24(1); **ST3**/ST10/ST14/ST24(1); **ST3**/ST31(1); **ST4**/ST24(1); **ST4**/ST24/ST31(1); **ST4**/ST10/ST14/ST21/ST24/ST25/ST26(1); ST10/ST14(2); ST10/ST14/ST23/ST24(1); ST10/ST14/ST24(7); ST10/ST14/ST31 (1); ST10/ST23/ST24/ST31(1); ST10/ST14/ST21(1); ST10/ST14/ST21/ST23/ST24(2); ST10/ST14/ST21/ST24/ST30(3); ST10/ST14/ST21/ST23/ST24/ST30(3); ST10/ST14/ST23/ST24/ST30(2); ST10/ST14/ST24/ST31(1); ST10/ST30(1); ST10/ST31(4); ST14/ST21/ST23(1);ST10/ST21/ST24(2); ST10/ST23(2); ST10/ST23/ST24(2); ST10/ST24(8); ST10/ST24/ST26(1); ST10/ST24/ST30(1); ST10/ST24/ST31(3); ST14(1); ST14/ST24(3); ST24(4); ST24/ST30(1); ST24/ST31(5); ST31(2)

^a^ No gender and age information available for 1 WTD.

**Table 3:** *Blastocystis* subtypes identified in white-tailed deer (WTD) from Maryland (USA) including number of samples in which each subtype was identified and number of unique sequences among each subtype.

*Blastocystis* Subtype	No. of *Blastocystis*-Positive Samples	Percentage of Positive WTD	No. of Unique Sequences within Subtype
ST1	1	1.4	2
ST3	2	2.8	2
ST4	3	4.2	2
ST10	51	71.8	14
ST14	30	42.3	3
ST21	14	19.7	3
ST23	14	19.7	1
ST24	55	77.5	3
ST25	1	1.4	1
ST26	2	2.8	3
ST30	11	15.5	3
ST31	19	26.8	1

## Data Availability

All relevant data are within the article and its additional files. All raw fastq files were deposited to the NCBI sequence read archive under accession number SRR14607063-SRR14607133. The sequences data were submitted to the GenBank database under the accession numbers MZ267636-MZ267679.

## Supplementary Materials

The following are available online at https://www.mdpi.com/article/10.3390/microorganisms9061343/s1. Supplementary Table S1. Pairwise distances between *Blastocystis* subtypes full-length *SSU* rRNA gene sequences showing number of base substitutions per site. Analyses were conducted using the Kimura 2-parameter model and included 70 nucleotide sequences. There were a total of 1950 positions in the final dataset. Supplementary Table S2. Pairwise distances between *Blastocystis* subtypes barcoding region sequences showing number of base substitutions per site. Analyses were conducted using the Kimura 2-parameter model and included 70 nucleotide sequences. There were a total of 590 positions in the final dataset. Supplementary Table S3. Pairwise distances between *Blastocystis* subtypes Santin region sequences showing number of base substitutions per site. Analyses were conducted using the Kimura 2-parameter model and included 70 nucleotide sequences. There were a total of 571 positions in the final dataset.
